# Sex differences in risk and heritability estimates on primary knee osteoarthritis leading to total knee arthroplasty: a nationwide population based follow up study in Danish twins

**DOI:** 10.1186/s13075-016-0939-8

**Published:** 2016-02-11

**Authors:** Søren Glud Skousgaard, Axel Skytthe, Sören Möller, Søren Overgaard, Lars Peter Andreas Brandt

**Affiliations:** Department of Occupational and Environmental Medicine, Odense University Hospital, 5000 Odense C, Denmark; Department of Orthopaedic Surgery and Traumatology & Orthopedic Research Unit, Institute of Clinical Research, University of Southern Denmark, 5000 Odense C, Denmark; Clinical Institute, University of Southern Denmark, 5000 Odense C, Denmark; Department of Epidemiology, Biostatistics and Biodemography, The Danish Twin Registry, University of Southern Denmark, 5000 Odense C, Denmark

**Keywords:** Knee osteoarthritis, Cumulative incidence, Twin study, Heritability, Total knee arthroplasty

## Abstract

**Background:**

Symptomatic knee osteoarthritis is a highly age and sex associated complex disease. Little is known about the causes behind this age and sex associated increase, or if genetic and environmental factors impacts differently by gender. Our study examined the risk and heritability of primary knee osteoarthritis leading to total knee arthroplasty and whether these differences were attributable to sex and age differences in heritability.

**Methods:**

All twins of known zygosity from The Danish Twin Register alive in 1997 were examined in a nationwide population based follow-up study collecting information on all twins recorded in The Danish Knee Arthroplasty from 1997 to follow-up in 2010. Our main outcomes were the cumulative incidence, probandwise concordance rates, heritability, within pair correlations in monozygotic and dizygotic twin pairs and the genetic and environmental influence estimated in models taking into account that individuals may not have had a total knee arthroplasty at follow up.

**Results:**

92,748 twins were eligible for analyses and 576 twins had a record of primary knee osteoarthritis in The Danish Knee Arthroplasty Register at follow-up comprising 358 female and 218 male twin cases. The risk increased particular after the age of 50 years displaying significant sex differences in the elderly. In the sex stratified analyses a discrete genetic component was found in females, but in males no genetic component could be detected. In both genders common and unique environmental factors were highly significant. In the sex-adjusted analysis an additive genetic component of 18 % (0; 62), a shared environmental component of 61 % (25; 97) and an individual environmental component of 21 % (6; 36) accounted for the variation in liability to primary total knee arthroplasty.

**Conclusion:**

The risk of primary total knee arthroplasty increases significantly after the age of 50 years, in particular in females, displaying significant sex differences in the elderly. After sex-adjustment 82 % of the variation in liability to primary total knee arthroplasty was attributable to common and unique environmental factors; the remaining 18 % of this variation was attributable to additive genetic factors indicating a pivotal impact of environmental factors on this disease.

## Background

Osteoarthritis (OA) is the most frequent joint disorder in western societies, and will presumably develop into one of the top-ranking causes of physical functional impairment and disability, including work-related disorders and absenteeism, during the next one or two decades [1–3]. Knee OA is a highly age and sex-associated prevalent and complex disease, with a substantial impact on the quality of life from pain and physical immobility, and consequently a significant limitation in social and working life [4]. Additionally, the co-morbidity associated with symptomatic knee OA is subject to concern because in a recent study an increased all-cause and disease-specific mortality has been reported in patients with symptomatic and radiographic hip and knee OA [5]. The prevalence of radiographic knee OA from the age of 50 years onwards ranges between 20 and 44 %, higher in females than in males [6–8]. However, the lifetime risk of symptomatic knee OA has recently been reported to be as high as 46.8 % and 39.8 % in females and males, respectively; in obese individuals, the lifetime risk of symptomatic knee OA was reported to be high as 60.5 % [9].

Knee OA is a complex and multifactorial disease caused by environmental and genetic factors. Environmental risk factors frequently referred to include body mass index (BMI) [10, 11], previous knee injury [12–14] and knee-straining work tasks [15, 16]. However, few studies into the genetic influence on knee OA have been published. A moderate genetic contribution to radiographic knee OA in healthy female twins has been reported in two cross-sectional studies with heritability estimates of 39 % and 37 % respectively [17, 18]. Additionally, the progression of radiographic knee OA in females has previously been reported to be under genetic influence [19]. These studies, however, examined non-symptomatic radiographic knee OA in females only. Consequently, little is known of the genetic influence in males, or whether genetic factors affect males and females differently or, indeed, whether genetic factors differ between radiographic and symptomatic knee OA. In a previously published sibling study including both genders listed for total knee arthroplasty (TKA), an increased risk in siblings to index cases after adjustment for sex, age, knee pain, BMI, Heberden’s nodes and meniscectomy was reported with odds ratios of 2.9 and 1.7 for tibiofemoral and patellofemoral OA, respectively [20]. In a previously published twin study, based on self-reported physician-diagnosed OA, the authors could not detect a genetic influence on OA (finger, hip, knee and ankle) in males, leaving common and unique environmental factors as the best model fit; however, in women an additive genetic component of 44 % in OA liability (finger, hip, knee and ankle) was reported [21]. However, the issue of OA in different joints being caused by common genetic processes or the genetic influence being joint site specific has previously been stressed as highly important for the design of future studies examining the nature of the genetic contribution to OA [18].

Studies defining knee OA cases from radiographic findings with or without symptoms may encounter some difficulties in defining their cases because disease severity varies and the correlation between clinical presentation and radiographic findings in OA generally is inconsistent [6–8, 22]. For reasons already mentioned we decided to use a twin who had undergone a TKA as a case, because this case definition represents a well-defined outcome and a heavy and significant disease burden contrary to cases based on conventional radiographic examination [22].

Accordingly, the objectives of this study were to examine the probability and heritability of primary OA of the knee leading to TKA, both sex stratified and sex adjusted, in a competing risk setting by means of the cumulative incidence function (CIF), biometric modelling, the age-related proband-wise concordance rates and cumulative heritability.

## Methods

**Fig. 1:**
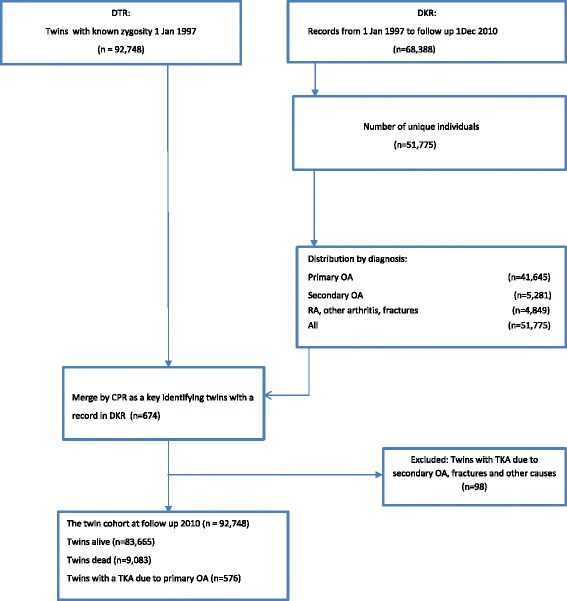
Flowchart of the participating twins. *CPR* Danish Personal Registration Number, *DKR* Danish Knee Arthroplasty Register, *DTR* Danish Twin Register, *OA* Osteoarthritis, *TKA* Total Knee Arthroplasty

Our study participants were selected from the Danish Twin Register (DTR). The DTR was established in 1954 and comprises more than 170,000 twins born in Denmark since 1870. The completeness of the twin ascertainment is high; after adjustment for infant mortality, 90 % of the twins born before and up to 1968 have been ascertained, with complete ascertainment of all live-born twins since 1968 [23, 24]. Zygosity of same-sex twins is assessed by a four-item questionnaire on the similarity of the two twins, which will classify their zygosity correctly in 95 % of all same-sex twin pairs [25]. All twins in the DTR alive at 1 January 1997 comprised the study cohort. Information on sex, date of birth, zygosity and vital status was used. The Danish Knee Arthroplasty Register (DKR) is a nationwide population-based health register established in 1997 and holds information on TKA surgery performed on a national basis. The register holds information on sex, date of birth, the WHO International Classification of Diseases version 10 (ICD10) diagnosis, being that which indicated the surgical treatment, and the date of surgery. The completeness of registration is 90–92 % based on annual reports and thus the DKR provides a sound basis for large population-based epidemiological studies [26, 27]. Using the unique Danish personal identification number (CPR) as a key for linkage, all Danish twins with a TKA and ICD10 diagnoses of M170 and M171 for the period of 1 January 1997–1 December 2010 were identified (Fig. 1). Information on sex, date of birth, the ICD10 diagnoses M170 and M171, and the date of surgery from the period of 1 January 1997–1 December 2010 were used. Our final study cohort comprised monozygotic (MZ), same-sex dizygotic (SS-DZ) and opposite-sex dizygotic (OS-DZ) twins, and a case was defined as a twin who had undergone a TKA due to primary knee OA independent of co-twin status.

### Ethical approval

The study was reviewed and approved by The Regional Research Ethical Committees of Southern Denmark and The Danish Data Protection Agency, and permission was granted to use the relevant data from the Danish Hip Arthroplasty Register and the DTR.

### Statistical analyses

Our objectives were to estimate the probability and heritability of TKA due to primary knee OA. To challenge these aims we implemented a time-to-event methodology in analysing our twin data taking into account the presence of right censoring and the competing risk of death in the study population.

**Fig. 2:**
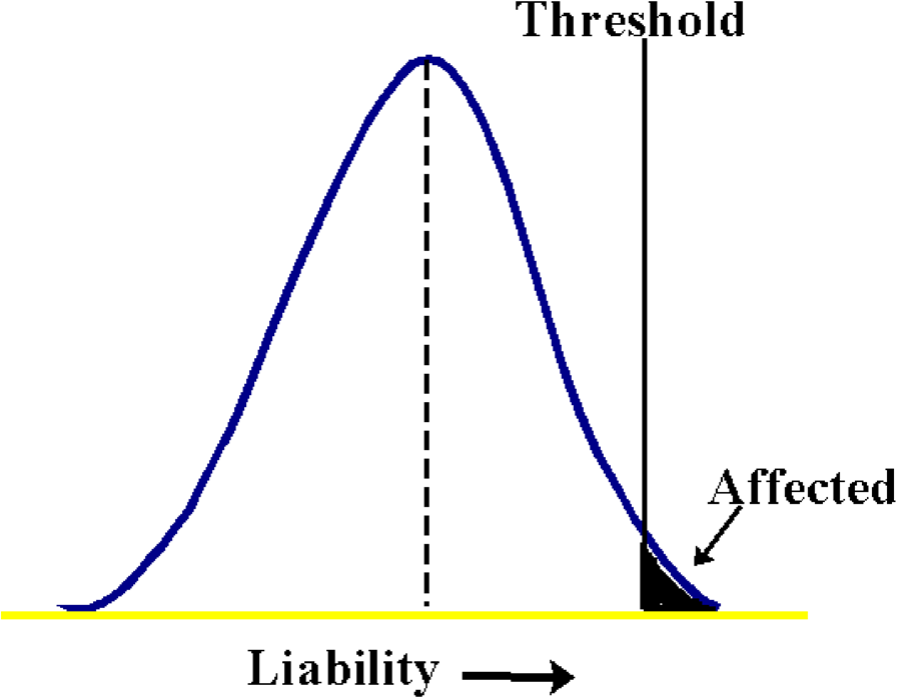
The threshold model

In the classical twin design (CTD), MZ twins are assumed to have identical genotypes, whereas DZ twins on average share one-half of their segregating genes as ordinary siblings. If a greater phenotypic similarity in MZ twins compared with that of DZ twins is observed, a genetic influence on the disease in question can be inferred [28]. The similarity in MZ and DZ twin pairs with a TKA was assessed by means of case-wise and proband-wise concordance rates and tetrachoric correlation coefficients. These concordance rates reflect the probability of one twin having the disease in question conditional that the co-twin is affected, and when all concordant pairs are doubly ascertained the proband-wise concordance rate equals that of the case-wise concordance rate [28]. The impact of environmental and genetic effects on a specific disease is reflected in the twin resemblance for liability to the disease expressed as correlations. In the case of a dichotomous outcome this resemblance is expressed as tetrachoric correlations and is estimated under the multifactorial threshold model assumption, which assumes that there is an underlying normally distributed liability to disease. The disease becomes manifest when an individual exceeds a threshold on the liability distribution corresponding to the overall prevalence of the disease [28, 29]. The threshold model is graphically displayed in Fig. 2.

Variation of a trait in a twin population can be separated into genetic factors, additive genetic (A) and dominant genetic (D), and environmental factors, shared environment (C) and unique environment (E) to the individual. Models containing combinations of these variance components can be constructed based on the known underlying correlation structure in a twin population [28].

To estimate the probability or risk of TKA we implemented the CIF, which takes the occurrence of competing risks into account [30–32]. If an individual is at risk of experiencing more than one event, each affecting the other, these events are called competing risks. In a population of older individuals with a long-term follow-up, the risk of knee OA leading to TKA and death become competing risks. We used the CIF to estimate the probability of undergoing a TKA because of knee OA adjusted for the competing risk of death by means of the Aalen–Johansen estimator. This method is based on the Markov illness death model with states ‘healthy’, ‘diseased’ and ‘dead’, where the transition probabilities describe the probability that a healthy individual at a later time will be diseased or dead [33].

Bivariate probit modelling in twin data is a new approach for analysing genetic and environmental factors on knee OA leading to TKA, and takes into account the presence of right censoring and competing risks in the data [34]. We implemented a weighted time regression analysis based on the inverse probability weighting (IPW) technique which is used to account for unbalanced sampling probabilities; weighting by the inverse of the probability of being sampled allows each observation to statistically represent the sampled non-case individuals as cases in the population [35, 36].

In a time-to-event analysis with competing risks, only a proportion of the cases are known at follow-up because individuals appearing as non-cases may become cases prospectively at a time we do not know, or are lost to follow-up. This is termed right censoring and we used liability-threshold modelling with IPW to analyse the twin data [29, 34–36].

Our biometric model fitting included saturated models and models composed of the variance components ACE, ADE, AE, DE and CE, both sex stratified and sex adjusted. For comparisons between models, a log likelihood ratio test and Akaike’s Information Criterion (AIC) were used. However, we also included the broad sense heritability based on the polygenetic liability-threshold model, which is a measure of the proportion of the variance in liability to a disease caused by additive and non-additive genetic effects [29].

The analyses were carried out in bivariate probit models for twin data implemented in the R Mets package (https://cran.r-project.org/web/packages/mets/index.html) and using the methods of estimating the proband-wise concordance function for competing risks data [37]. For comparisons between groups, a two-tailed *t* test or chi-square test was used as appropriate. *p* ≤0.05 was considered significant, and confidence intervals (CIs) were expressed as the 95 % CI. Calculations were carried out in the statistical software R and Stata11 (StataCorp LP, 4905 Lakeway Drive, College Station, Texas 77845-4512, USA).

## Results

Figure 1 displays the flow of the participating twins. The twin cohort comprised 92,748 twins, with 674 twins recorded in the DKR of which 576 had undergone a TKA due to primary knee OA at follow-up. Table 1 presents summary statistics from the two registers.

**Table 1:** Distribution of TKA by register, zygosity and sex

Distribution by register, sex and TKA	DTR	DKR	
Number	92,748	51,775	
Females	46,171	32,076	
Males	46,577	19,699	
Record in DKR	674^a^ (85)	41,645 (81)	
Females	349 (61)	26,719 (62)	
Males	227	15,111	
Mean age (years)	67.1 (39–94)	67.6 (17–100)	
Females	67.7 (40–94)	68.3 (17–100)	
Males	66.3 (39–88)	66.3 (21–96)	
Distribution by zygosity, sex and TKA	Males	Females	Total by sex
MZ with TKA	39	82 (68)	121
MZ without TKA	9,739	10,135 (51)	19,874
DZ with TKA	103	157 (60)	260
DZ without TKA	17,727	16,691 (48)	34,418
OS with TKA	85	110 (56)	195
OS without TKA	18,884	18,996 (50)	37,880
Total	46,577	46,171 (50)	92,748

Data presented as number, number (percentage) or mean (range)

^a^Primary TKA in 576 twins

*DKR* Danish Knee Arthroplasty Register, *DTR* Danish Twin Register, *DZ* dizygotic, *MZ* monozygotic, *OS* opposite sex, *TKA* total knee arthroplasty

### Tetrachoric correlations

Table 2 presents tetrachoric correlations in the same-sex and opposite-sex twin pairs where both twins were alive at entry. We found no significant differences between male MZ and DZ twin pairs contrary to female MZ and DZ twin pairs, indicating a lack of genetic component in males but detectable in females. The test for within-pair independence for TKA in MZ males is borderline significant (*p* = 0.05) compared with that of DZ male pairs (*p* <0.0001), further indicating that a genetic influence in males may be rather insignificant. However, the correlation in DZ male pairs was markedly higher than that of the MZ male pairs, indicating a significant influence from common environmental factors in males.

**Table 2:** Concordant and disconcordant pairs and tetrachoric correlations by zygosity in twin pairs where both twins in the pair were alive at entry

Zygosity	Concordant TKA due to OA pairs (*n*)	Discordant TKA due to OA pairs (*n*)	Concordant pairs without OA pairs (*n*)	Tetrachoric correlation (rho) (*p* value^a^)
MZ males	1	29	4509	0.45 (0.05)
DZ males	4	72	8176	0.51 (<0.0001)
MZ females	10	52	4701	0.73 (<0.0001)
DZ females	6	122	7575	0.42 (<0.0001)
OS-DZ	3	158	17,490	0.36 (0.004)

^a^Test of within-pair independence for TKA due to OA

*DZ* dizygotic, *MZ* monozygotic, *OA* osteoarthritis, *OS-DZ* opposite-sex dizygotic, *rho* tetrachoric correlation coefficient, *TKA* total knee arthroplasty

### Cumulative incidence function

**Fig. 3:**
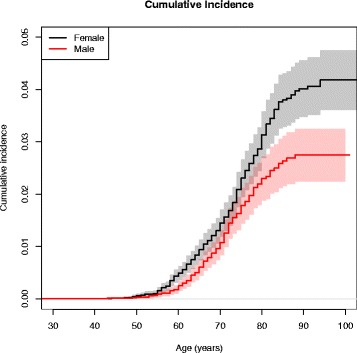
Cumulative incidence for TKA in males (*red dashed line*) and females (*black dashed line*)

Figure 3 shows the cumulative incidence for TKA due to primary knee OA. An increasing risk was found from the age of 50 years in both genders; peaking to 2.5/100 individuals in males and 4/100 individuals in females at 85 years of age.

### Proband-wise concordance rates on age

**Fig. 4:**
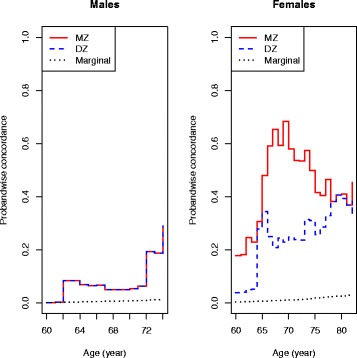
Proband-wise concordance rate of TKA by age and sex. *DZ* dizygotic, *Marginal* background incidence in the genetically unrelated twin population, *MZ* monozygotic

In Fig. 4 the proband-wise concordance rates including censoring are displayed separately for men and women. In males, no difference in MZ and DZ twin pairs was observed; indicating that the genetic influence in males was not detectable or present. In females, a MZ/DZ twin pair difference is closing in by age, indicating a decreasing genetic influence in females by age; however the 95 % CIs did overlap.

### Prevalences, tetrachoric correlations and case-wise concordance rates

Equal prevalence in MZ and DZ twin pairs in all models was observed; however, in males the saturated model (all co-variances are treated as free parameters) did not support the existence of a genetic component, as both the case-wise concordance rates and tetrachoric correlations did not differ between MZ and DZ twin pairs. In females this difference was detectable, but not significant, albeit favouring a broad sense heritability of 6 % (0; 73). In the sex-adjusted saturated model the differences in case-wise concordance rates and tetrachoric correlations coefficients between MZ and DZ twin pairs were notable, favouring a broad sense heritability of 18 % (0; 62) (see Tables 3, 4 and 5).

**Table 3:** Heritability and biometric modelling in males with knee OA

Model in males	MZ prevalence	DZ prevalence	MZ rho	DZ rho	MZ cwc	DZ cwc	H	Variance component estimates				Chi-square test statistics				
								A	D	C	E	LL	–2ln	df	*p* value	AIC
Sat	0.02 (0.01–0.02)	0.02 (0.01–0.02)	0.66 (0.03–0.92)	0.71 (0.39–0.87)	0.26 (0.05–0.70)	0.30 (0.13–0.55)	0 (0.00–0.89)	–	–	–	–	–3387.1	0.021	1	Compare CE, *p* = 0.65	6780.14
ACE	0.02 (0.01–0.02)	0.02 (0.01–0.02)	0.7 (0.42–0.85)	0.7 (0.42–0.85)	0.29 (0.14–0.51)	0.29 (0.14–0.51)	0	0	–	0.69 (0.48–0.90)	0.31 (0.1–0.52)	–3387.2	25.8	1	Compared AE, *p* <0.0001	6780.35
ADE	0.02 (0.01–0.02)	0.02 (0.01–0.02)	0.79 (0.45–0.93)	0.39 (0.28–0.50)	0.39 (0.18–0.66)	0.10 (0.07–0.16)	0.79 (0.57–1.0)	0.79 (0.57–1.0)	0	–	0.21 (0.0–0.43)	–3400.1	0	1	Compare DE, *p* = 1.0	6806.14
AE	0.02 (0.01–0.02)	0.02 (0.01–0.02)	0.79 (0.45–0.93)	0.39 (0.28–0.50)	0.39 (0.18–0.66)	0.10 (0.07–0.16)	0.79 (0.57–1.0)	0.79 (0.57–1.0)	–	–	0.21 (0.0–0.43)	–3400.1	25.8	1	Compare Sat, *p* <0.0001	6804.14
**CE**	**0.02 (0.01–0.02)**	**0.02 (0.01–0.02)**	**0.69 (0.42–0.85)**	**0.69 (0.42–0.85)**	**0.29 (0.14–0.51)**	**0.29 (0.14–0.51)**	**–**	**–**	**_**	**0.69 (0.48–0.90)**	**0.31 (0.1–0.52)**	**–3387.2**	**0**	**1**	**Compare ACE,** ***p*** **= 1.0**	**6778.35**
DE	0.02 (0.01–0.02)	0.02 (0.01–0.02)	0.76 (0.38–0.92)	0.19 (0.13–0.25)	0.36 (0.14–0.66)	0.04 (0.03–0.06)	0.76 (0.51–1.0)	–	0.76 (0.51–1.0)	–	0.24 (0.00–0.49)	–3414.6	29.1	1	Compare ADE, *P* <0.0001	6833.24

*A* additive genetic, *AIC* Akaike’s Information Criterion, *C* common environment, *cwc* case-wise concordance rate, *D* dominant genetic, *df* degrees of freedom, *DZ* dizygotic, *E* unique environment, *H* broad sense heritability, *LL* = log likelihood of model, –2ln = Likelihood Ratio chi-square test, *MZ* monozygotic, *rho* tetrachoric correlation coefficient, *Sat* saturated model, *OA* osteoarthritis

The CE model in bold displayes the best model fit by the AIC. Fixing the additive genetic component at zero produced no worse fit (p=1.0)

**Table 4:** Heritability and biometric modelling in females with knee OA

Model in females	MZ prevalence	DZ prevalence	MZ rho	DZ rho	MZ cwc	DZ cwc	H	Variance component estimates				Chi-square test statistics				
								A	D	C	E	LL	–2ln	df	*p* value	AIC
Sat	0.03 (0.03–0.04)	0.03 (0.03–0.04)	0.77 (0.53–0.90)	0.74 (0.48–0.88)	0.42 (0.25–0.62)	0.39 (0.22–0.60)	0.06 (0.0–0.58)	–	–	–	–	–5877.6	34.2	1	Compare AE, *p* <0.0001	11,552.2
**ACE**	**0.03 (0.03–0.04)**	**0.03 (0.03–0.04)**	**0.77 (0.53–0.90)**	**0.74 (0.48–0.88)**	**0.42 (0.25–0.62)**	**0.39 (0.22–0.60)**	**0.06 (0.0–0.58)**	**0.06 (0.0–0.58)**	**–**	**0.71 (0.29–1.00)**	**0.23 (0.06–0.41)**	**–5877.6**	**34.2**	**1**	**Compare AE,** ***p*** **<0.0001**	**11,552.2**
ADE	0.03 (0.03–0.04)	0.03 (0.03–0.04)	0.80 (0.62–0.91)	0.40 (0.33–0.47)	0.46 (0.30–0.63)	0.15 (0.12–0.19)	0.80 (0.67–0.94)	0.80 (0.67–0.94)	0	–	0.20 (0.06–0.33)	–5894.8	0	1	Compare AE, *p* = 1.0	11,575.6
AE	0.03 (0.03–0.04)	0.03 (0.03–0.04)	0.80 (0.62–0.91)	0.40 (0.33–0.47)	0.46 (0.30–0.63)	0.15 (0.12–0.19)	0.80 (0.67–0.94)	0.80 (0.67–0.94)	–	–	0.20 (0.06–0.33)	–5894.8	23.3	1	Compare Sat, *p* <0.0001	11,573.6
CE	0.03 (0.03–0.04)	0.03 (0.03–0.04)	0.76 (0.60–0.86)	0.76 (0.60–0.86)	0.41 (0.28–0.55)	0.41 (0.28–0.55)	–	–	_	0.76 (0.63–0.88)	0.24 (0.12–0.37)	–5877.7	0.26	1	Compare ACE*, p* = 0.61	11,553.5
DE	0.03 (0.03–0.04)	0.03 (0.03–0.04)	0.79 (0.59–0.90)	0.20 (0.16–0.24)	0.45 (0.38–0.63)	0.08 (0.06–0.09)	0.79 (0.64–0.94)	–	0.79 (0.64–0.94)	–	0.19 (0.05–0.33)	–5912.5	35.5	1	Compare ADE, *p* <0.0001	11,606.5

*A* additive genetic, *AIC* Akaike’s Information Criterion, *C* common environment, *cwc* case-wise concordance rate, *D* dominant genetic, *df* degrees of freedom, *DZ* dizygotic, *E* unique environment, *H* broad sense heritability, *LL* = log likelihood of model, –2ln = Likelihood Ratio chi-square test, *MZ* monozygotic, *rho* tetrachoric correlation coefficient, *Sat* saturated model, *OA* osteoarthritis

The ACE model in bold displayes the best model fit by the AIC. Fixing the common environmental component C at zero produced a significantly worse fit (*p* <0.0001)

**Table 5:** Biometric models and chi-square test statistics, sex adjusted

Model	MZ prevalence	DZ prevalence	MZ rho	DZ rho	MZ cwc	DZ cwc	H	Variance component estimates				Chi-square test statistics				
								A	D	C	E	LL	–2ln	df	*p* value	AIC
Sat	0.03 (0.03–0.04)	0.03 (0.03–0.04)	0.79 (0.59–0.90)	0.70 (0.50–0.83)	0.44 (0.28–0.62)	0.35 (0.22–0.51)	0.18 (0.00–0.62)	–	–	–	–	–10014.6	3.87	1	Compare CE, *p* = 0.05	20,037.10
**ACE**	**0.03 (0.03–0.04)**	**0.03 (0.03–0.04)**	**0.79 (0.59–0.90)**	**0.70 (0.50–0.83)**	**0.44 (0.28–0.62)**	**0.35 (0.22–0.51)**	**0.18 (0.0–0.62)**	**0.18 (0.0–0.62)**	**–**	**0.61 (0.25–0.97)**	**0.21 (0.06–0.36)**	**–10014.5**	**44.6**	**1**	**Compare AE,** ***p*** **<0.0001**	**20,037.10**
ADE	0.03 (0.03–0.04)	0.03 (0.03–0.04)	0.83 (0.68–0.91)	0.41 (0.36–0.47)	0.49 (0.35–0.64)	0.16 (0.13–0.19)	0.83 (0.72–0.94)	0.83 (0.72–0.94)	0	–	0.17 (0.06–0.28)	–10036.8	0	1	Compare AE, *p* = 1.0	20,081.66
AE	0.03 (0.03–0.04)	0.03 (0.03–0.04)	0.83 (0.68–0.91)	0.41 (0.36–0.47)	0.49 (0.35–0.64)	0.16 (0.13–0.19)	0.83 (0.72–0.94)	0.83 (0.72–0.94)	–	–	0.17 (0.06–0.28)	–10036.8	34.3	1	Compared ACE, *p* <0.0001	20,079.66
CE	0.03 (0.03–0.04)	0.03 (0.03–0.04)	0.74 (0.61–0.83)	0.74 (0.61–0.83)	0.39 (0.29–0.51)	0.39 (0.29–0.51)	–	–	–	0.74 (0.63–0.85)	0.26 (0.15–0.37)	–10016.5	3.87	1	Compare ACE, *p* = 0.05	20,038.97
DE	0.03 (0.03–0.04)	0.03 (0.03–0.04)	0.82 (0.66–0.91)	0.20 (0.17–0.23)	0.48 (0.33–0.63)	0.08 (0.07–0.09)	0.81 (0.70–0.94)	–	0.82 (0.70–0.94)	–	0.18 (0.06–0.30)	–10067.7	61.7	1	Compare ADE, *p* <0.0001	20,141.35

*A* additive genetic, *AIC* Akaike’s Information Criterion, *C* common environment, *cwc* case-wise concordance rate, *D* dominant genetic, *df* degrees of freedom, *DZ* dizygotic, *E* unique environment, *H* broad sense heritability, *LL* = log likelihood of model, –2ln = Likelihood Ratio chi-square test, *MZ* monozygotic, *rho* tetrachoric correlation coefficient, *Sat* saturated model.

The ACE model in bold displayes the best model fit by the AIC. Fixing the common environmental component C at zero produced a significantly worse fit (*p* <0.0001).

### Biometric modelling

The first model in Tables 3, 4 and 5 displays the broad sense heritability estimates reflecting the proportion of the variance attributable to genetic factors. In males, the best fitting model was the CE model of common and individual environmental factors; fixing A at 0 in the ACE model did not change the fit at all (*p* = 1.0) and the CE gave the best fit by the AIC. In females, the ACE model provided the best fit to the data; setting A at 0 in the ACE model did not produce a significantly worse fit (*p* = 0.61), but the ACE model gave the best fit by the AIC. In the sex-adjusted saturated model the DZ correlation was markedly larger than half that of the MZ correlation, underlining the presence of a common environmental component. Fixing C at 0 in the sex-adjusted ACE model produced a significantly worse fit in the AE model (*p* <0.0001). Setting A at 0 in the sex-adjusted ACE model produced a significant worse fit in the CE model (*p* = 0.05). Excluding a genetic component by comparing the CE with the saturated model likewise produced a significant worse fit (*p* = 0.05) and the sex-adjusted ACE model gave the best fit comparing the alternative models AE, ADE, DE and CE by the AIC. This model produced an additive genetic component of 18 % (0; 62) and a highly significant common environmental component of 61 % (25; 97), with a unique environmental component accounting for 21 % (6; 36) of the population liability for TKA due to primary knee OA.

Both the sex-stratified and sex-adjusted best model fit provided ambient evidence of a highly significant common environmental influence. Indeed, the observed difference in DZ–MZ correlations—that is, the DZ correlation was markedly larger than half that of the MZ correlation—strengthened and emphasised the presence of a common environmental component favouring the ACE and CE models in females and males, respectively.

### Heritability on age

**Fig. 5:**
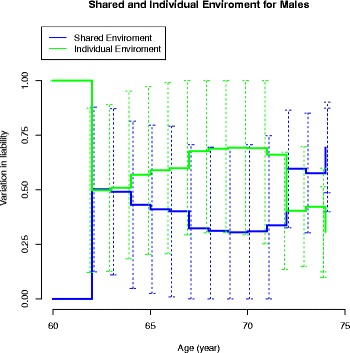
Heritability in males – the CE model

**Fig. 6:**
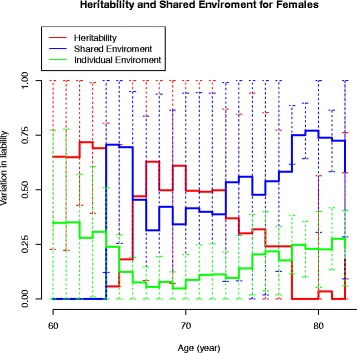
Heritability in females – the ACE model

**Fig. 7:**
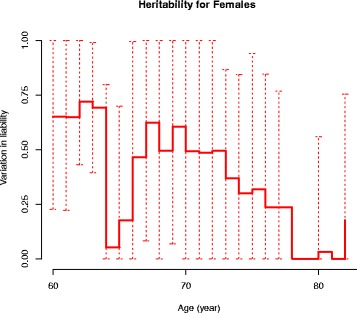
Heritability on age in females

The age-associated changes in the variance components in the CE and ACE models are displayed in Fig. 5 for males and in Figs. 6 and 7 for females, respectively. In males, the common and individual environmental influences remain high from 60 to 70 years of age. In females, the genetic influence decreased with increasing age to disappear at age around 80 years. In Fig. 7 the heritability on age in females is displayed with an estimate of 40 % at age 65–66 years.

## Discussion

To our knowledge this is the first twin study of primary knee OA leading to TKA, including both sexes, as well as the same-sex and opposite-sex twin pairs. We could not estimate a genetic component in males, inferring knee OA in males as primarily an environmental disease. In women an additive genetic influence was detectable, accounting for 6 % of the variation in population liability for knee OA leading to TKA. However, a very interesting finding was the robust significance of the common environmental component in both the sex-stratified and sex-adjusted analyses. In terms of family factors, comprising common genes and common environment, 79 % of the variation in liability to knee OA in females was attributable to these factors; however, 69 % of the variation in liability to knee OA in males was attributable to common environmental factors alone. In our sex adjusted analysis the additive genetic component accounted for 18 % of the variation in the population liability to TKA due to primary OA leaving 82 % of this variation attributable to environmental factors attributable to 82 % of the variation in population liability for TKA due to primary OA. Consequently, the main findings in our study are that in both males and females knee OA is primarily an environmental disease and the impact of genetic factors is of a lesser significance.

### Strengths

Our study has several strengths. Overall, twins are representative of the general population with respect to common multifactorial complex diseases [38]. Our results support this notion because we found the same distribution of sex and cases in twins as in non-twins in the DKR; 85 % of the twins had undergone a TKA compared with 82 % in the non-twin register population, and a same-sex distribution of 61–62 % females occurred in twin and non-twin subpopulations, respectively. Our data were collected from two Danish nationwide and population-based registers including a large twin population comprising all twins alive in 1997 and a follow-up term of 13 years. The completeness of in the DKR is approximately 90–92 % based on annual reports [26]. The DKR has been validated and found to provide a sound basis for large population-based epidemiological studies [27]. Further, the zygosity misclassification in the DTR of the same-sex twins is an acceptable 5 % [25].

By implementing the CIF we took into account that the occurrence of death and TKA due to primary OA by increasing age are competing events which might otherwise have biased our estimates if not adjusted for by overestimating the number of cases [32].

The cumulative incidence curves reflect the risk of TKA, separately in males and females, and are intuitively appealing and easy to understand. Further, the censored nature of the data collected from 1997 to 2010 was accounted for by applying a liability threshold model with right censoring. We defined a case as a twin who had undergone a TKA due to knee OA independent of co-twin status, because these patients represent a well-defined outcome contrary to cases based on conventional radiographic examination with or without symptoms [22]; studies defining knee OA cases from radiographic findings may encounter some difficulties in defining their cases as disease severity varies and the correlation between symptoms or clinical presentation and radiographic findings generally is poor [6–8, 22, 39]. Further, TKA is frequently the treatment of choice when symptoms cannot be controlled in any other way regardless of radiographic findings.

### Limitations

Our study has some important limitations. Some misclassification with respect to the proper diagnosis and zygosity may occur, but a certain misclassification of the diagnosis would be equally distributed between zygosity groups and would not have any predestined pattern (i.e. MZ twin pairs). The principle in the CTD is that if a larger phenotypic similarity is observed in MZ twin pairs compared with that of DZ twin pairs, a genetic influence on the disease in question can be inferred; however, we consider it unlikely that either misclassification or completeness should be subject to zygosity favouritism, hence this misclassification would be of the non-differential type. Adjustments for confounder effects in register-based studies are only feasible if the relevant information is included in the register in question. In this study we could not adjust for BMI and occupational exposures, as the two registers did not provide this information. However, studies on BMI as a risk factor for knee OA are numerous and conclusive [9, 10]. Our heritability estimates may be biased because we could not adjust for BMI, in particular whether BMI is considered to be influenced by shared environmental factors. The common environment includes shared family life with respect to upbringing and cohabitation, potentially inflicting a negative association between family factors and the risk of knee OA in later life. BMI is a highly heritable condition [40]; however, common environmental factors have previously been reported to influence BMI significantly in twin studies with sufficient sample size, pointing at a genotype × environment covariance (CV_GE_) [41, 42]. Regarding the high correlation pattern between BMI and knee OA, a CV_GE_ between knee OA susceptibility genes and common environmental influences on BMI is a likely assumption. Such a CV_GE_ relationship driven by shared environmental factors may mimic a common environmental influence at the expense of the additive genetic component. A CV_GE_ is a well appreciated bias in the CTD, as well as the difficulties in separating the common environmental component from an additive genetic effect, and is described in detail by Coventry and Keller [43, 44]. If parental phenotypes, more or less of genetic origin, modify the environment of their child or children, a CV_GE_ may be present. Consequently, the presence of a CV_GE_ will increase the DZ similarity relative to the MZ similarity and mimic a common environmental influence at the expense of an additive genetic influence, violating the equal environment assumption essential in the CTD [43, 44]. Hence, parental BMI susceptibility genes may rule or govern environmental exposure like diet and physical activity preferences, so that throughout life individuals at risk of knee OA may live in high calorie intake and low physical activity environments as they hang on to environments correlated with their genetic propensities [42]. Indeed, in a recent study by Reyes et al. [45], individuals from the lowest socio-economic status families had a significantly higher risk of knee OA, obesity accounting for 50 % of the excess risk. However, a weakness in the CTD is that it cannot discriminate the various sources of the shared environment, but for this purpose the extended twin-family design is well appreciated [28, 43, 44].

Studies on occupation and occupational exposure to knee-straining work tasks and the risk of symptomatic knee OA have primarily demonstrated a moderate association between kneeling work tasks and heavy lifting, and certain professions (i.e. farming and fishing) as risk factors for knee OA, but primarily in males [14–16, 22]. However, a recent study by Andersen et al. [46] demonstrated an increased risk of knee OA in female health care assistants.

### Context

In our sex-adjusted ACE model (censoring included) a heritability estimate of 18 % (0; 62) and a common environmental component of 61 % (25; 97) were found. To our knowledge, this is the first nationwide population-based twin study demonstrating a highly significant influence from common environmental factors in the liability for TKA due to primary knee OA. Few twin studies on knee OA have been published; however, our study indicates a non-significant additive genetic component of 6 % in women, which is much lower than the findings of Spector et al. [17] and MacGregor et al. [18] of 39 % and 37 % respectively. These latter studies were both cross-sectional in design and based on women with radiographic knee OA, a much younger population compared with our study. By design, these studies could not take into account the competing event of death that may influence the age-related occurrence of symptomatic knee OA and the resulting heritability estimates. Our lower estimate may partly be a consequence of not being able to adjust for confounders such as BMI, but our graphical presentation in Fig. 6 of the heritability in the ACE model in females indicates a higher genetic component in younger females, decreasing with increasing age to disappear at age around 80 years. In Fig. 7 the heritability on age in females is displayed with an estimate of 40 % at age 65. The drop in heritability at ages 64–65 years does not reflect a biological plausible course, but rather is caused by the difficulties in the CTD of separating the additive genetic component from that of the common environment as described previously.

That genetic influences on a trait may change with age has recently been described in a large twin-based follow-up study where the age-associated changes in bone mineral density were reported to be highly heritable in younger women, but the heritability decreased by increasing age to disappear after the age of 65 years [47]. In the sibling study on knee OA by Neame et al. [20] a heritability estimate of 62 % was reported, although the authors did recommend a cautious interpretation as they could only adjust for age.

We could not detect a genetic component in males. This is in line with the twin study by Kujala et al. [21] on hip, knee, hand and ankle OA, which failed to establish a genetic component in males. We have recently examined the risk and heritability of primary hip OA leading to total hip arthroplasty, implementing the same methodology as in the present study. We found a highly significant additive genetic component after adjustment for sex of 47 % and a common environmental component of 22 % of the variation in liability for total hip arthroplasty due to primary hip OA [48]. These findings indicate that the genetic and environmental influences differ by joint site and support the findings by MacGregor et al. [18] that hip and knee OA share less than 1 % common genes and that genetic and environmental factors impact differently in the two weight-bearing joints.

Knee OA pain may differ by sex, in that females tend to report higher pain score compared with males and a variety of socio-economic and mental factors may interact with knee OA pain [49, 50]. In our study, data on pain score, socio-economic factors or mental complaints were not available, leaving no possibility to adjust for these potential confounders or to examine a genetic influence on knee OA pain per se.

However, by including both MZ and SS-DZ as well as OS-DZ twin pairs, adjustment for gender was feasible and our heritability estimate reflects the influence from genetic, common and environmental factors on end-stage knee OA leading to TKA after adjustment for sex effects.

### Implications

The graphical presentation of the cumulative incidence provides a ready instrument assessing the risk of TKA and provides helpful insight to clinicians as well as to health care planners and occupational hygienists, because our study indicates that the influence from environmental factors on TKA risk is highly important in both genders. In western societies the populations are getting older and the proportion of older individuals is increasing rapidly, as is overweight and obesity. These factors increase the risk of knee OA, increase the health economic burden and increase the risk of death [1–5]. This development will need increasing focus and our study provides some evidence that intervention by clinical and public health measures, including preventive measures, should focus in particular on the family from childhood and adolescence to prevent increasing knee OA incidence and prevalence.

## Conclusion

Our study indicates that the impact of genetic factors on knee OA leading to TKA differ in females and males, but simultaneously also demonstrates the presence of a highly significant influence from common and unique environmental factors. In the sex-stratified model fitting, an additive genetic influence of 6 % in females was detected, but in males no genetic component was found. However, after sex adjustment the impact of environmental factors remained highly significant, leaving a moderate additive genetic influence of 18 % in the population liability for TKA. Further studies focusing on the discrimination of the various sources of the common environment in exposed families and individuals are needed, as well as studies on preventive measures.

### Data sharing

None.

## Abbreviations

A: Additive genetic
AIC: Akaike’s Information Criterion
BMI: Body mass index
C: Common environment
CI: Confidence interval
CIF: Cumulative incidence function
CTD: Classical twin design
CV_GE_: genotype × environment covariance
D: Dominant genetic
DKR: Danish Knee Arthroplasty Register
DTR: Danish Twin Register
DZ: Dizygotic
E: Unique environment
IPW: Inverse probability weighting
MZ: Monozygotic
M17.0: Primary gonarthrosis, bilateral
M17.1: Other primary gonarthrosis
OA: Osteoarthritis
OS-DZ: Opposite-sex dizygotic
rho: tetrachoric correlation coefficient
SS-DZ: Same-sex dizygotic
TKA: Total knee arthroplasty
ICD10: WHO International Classification of Diseases version 10
